# Supplementary material for: Determining subunit-subunit interaction from statistics of cryo-EM images: observation of nearest-neighbor coupling in a circadian clock protein complex
Source: Nat Commun. 2023 Sep 22;14:5907. doi: 10.1038/s41467-023-41575-1 (PMC10516925; doi:10.1038/s41467-023-41575-1)
Supplement: Supplementary file 1 — Supplementary Information [file 41467_2023_41575_MOESM1_ESM.pdf]

**Supplementary Information for "Determining subunit-subunit interaction from statistics of cryo-EM images: Observation of nearest-neighbor coupling in a circadian clock protein complex"**

Xu Han[1], Dongliang Zhang[1], Lu Hong[3], Daqi Yu[1], Zhaolong Wu[1], Tian Yang[1],
Michael Rust[4]*, Yuhai Tu [5]*, Qi Ouyang [1,2]*

*1 State Key Laboratory of Artificial Microstructure and Mesoscopic Physics, School of Physics, Peking University, Beijing 100871, China*

*2 Center for Quantitative Biology and Peking-Tsinghua Center for Life Sciences, AAIC, Peking University, Beijing 100871, China*

*3 Graduate Program in Biophysical Sciences, University of Chicago, Chicago IL 60637, USA*

*4 Departments of Molecular Genetics and Cell Biology and of Physics, University of Chicago, Chicago IL 60637, USA*

*5 IBM T. J. Watson Research Center, Yorktown Heights, NY 10598, USA*

# S1 Cryo-EM structure determination and data processing

| | KaiC-AA | KaiC-EE |
|---|---|---|
| **Data collection and processing** | | |
| Magnification | 105,000 | 105,000 |
| Voltage (kV) | 300 | 300 |
| Electron exposure (e–/Å$^2$) | 50 | 50 |
| Defocus range (μm) | -0.4 to -5.0 | -0.4 to -5.0 |
| Pixel size (Å) | 0.685 | 0.685 |
| Symmetry imposed | C6 | C6 |
| Initial particle images (no.) | 1,592,573 | 934,373 |
| Final particle images (no.) | 140,475 | 181,326 |
| Map resolution (Å) | 3.3 | 3.3 |
| FSC threshold | 0.143 | 0.143 |
| **Refinement** | | |
| Map sharpening *B* factor (Å$^2$) | -100 | -100 |
| **Model composition** | | |
| Non-hydrogen atoms | 22,026 | 23,298 |
| Protein residues | 2,748 | 2,904 |
| Ligands | 24 | 24 |
| **R.m.s. deviations** | | |
| Bond lengths (Å) | 0.006 | 0.003 |
| Bond angles (°) | 0.852 | 0.753 |
| **Validation** | | |
| MolProbity score | 1.65 | 1.67 |
| Clashscore | 3.60 | 4.99 |
| Rotamers outliers (%) | 0.00 | 0.04 |
| **Ramachandran plot** | | |
| Favored (%) | 91.72 | 93.84 |
| Allowed (%) | 8.28 | 6.16 |
| Outliers (%) | 0.00 | 0.00 |

Supplementary Table1: Cryo-EM data collection, refinement and validation statistics.

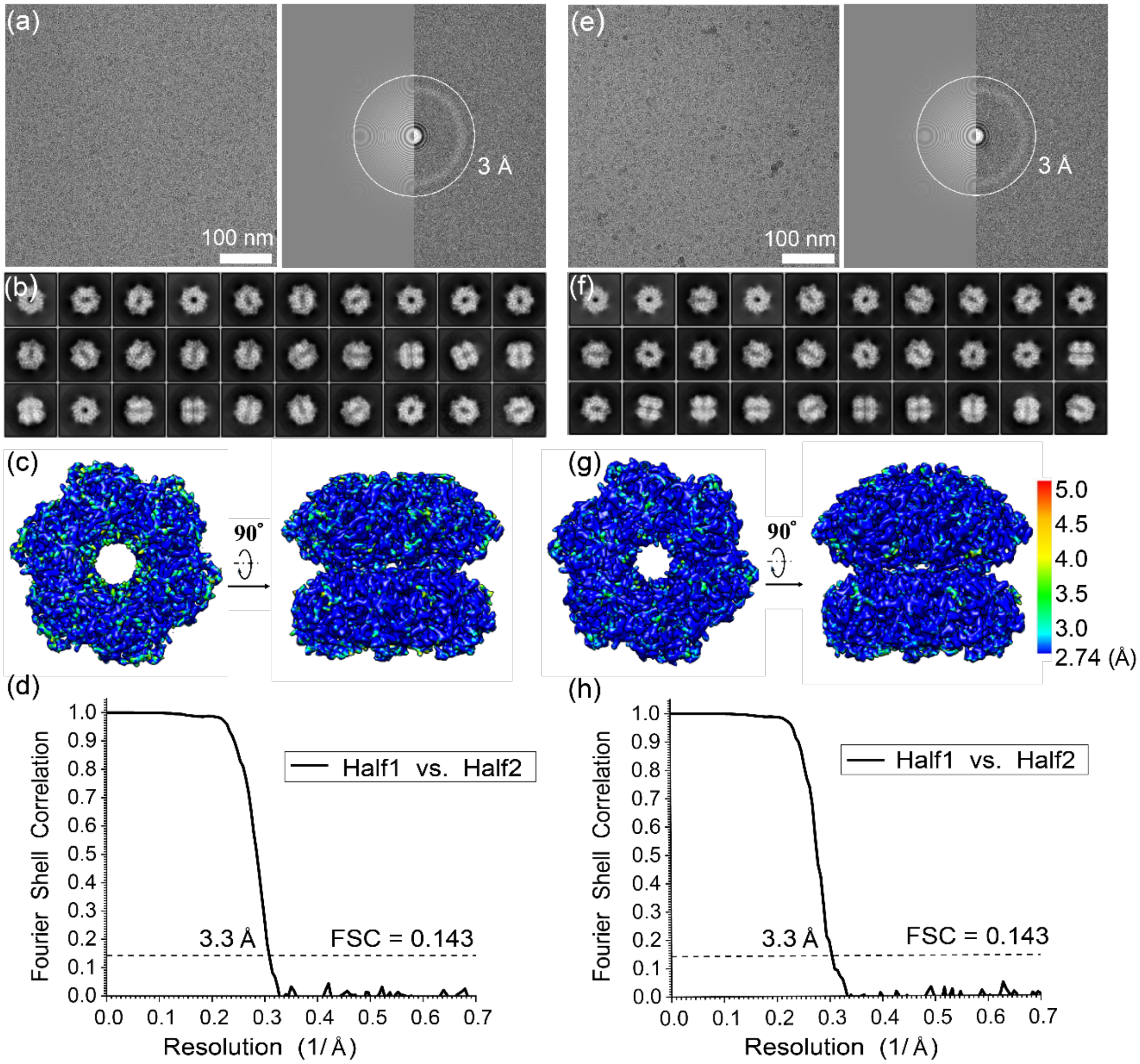


**Supplementary Figure 1: Cryo-EM structure determination of KaiC-AA and KaiC-EE.** On the left is a typical cryo-EM micrograph of KaiC-AA **(a)** and of KaiC-EE **(e)**, taken with a FEI Titan Krios G2 microscope equipped with the post-column Gatan BioQuantum energy filter connected to Gatan K2 Summit direct electron detector. Scale bar, 100 nm. On the right is the power spectrum evaluation corresponds to the left micrograph. (b) and (f) give gallery of unsupervised 2D class averages of KaiC-AA **(b)** and of KaiC-EE **(f)**. (c) and (g) provide local resolution estimation calculated by ResMap for KaiC-AA **(c)** and for KaiC-EE **(g)**. (d) and (h) show Fourier shell correlation (FSC) for two independently refined halves of KaiC-AA **(d)** and of KaiC-EE **(h)**. During the refinement we imposed C6 symmetry.

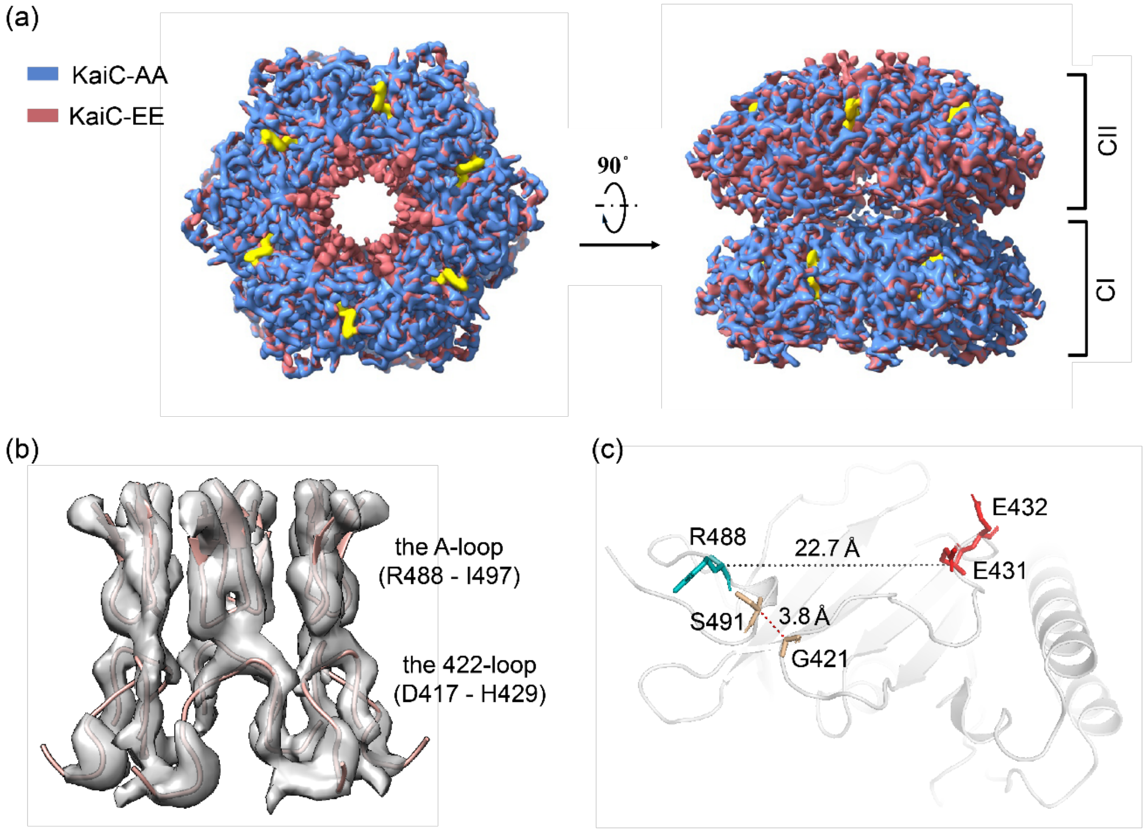


**Supplementary Figure 2: Comparison of KaiC-AA and KaiC-EE cryo-EM densities. (a)** Superimposition of KaiC-AA density (colored in royal blue) and KaiC-EE density (colored in red). **(b)** The difference map between KaiC-EE and KaiC-AA cryo-EM density is rendered as a transparent surface, superimposed with the cartoon representation of the atomic model of the A-loop (residue 488-497) and the 422-loop (residue 417-429). **(c)** A close-up view of this interacting-loops region in KaiC-EE CII domain (subunit A). Side chains of G421, E431, E432, R488, S491 and distance between G421 and S491 are labeled.

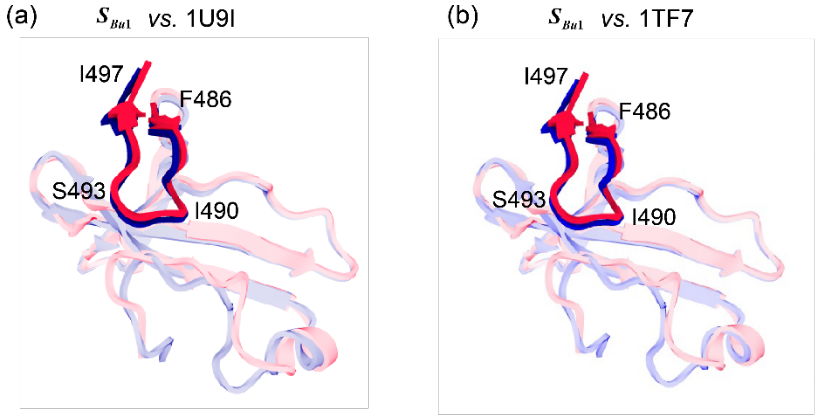


**Supplementary Figure 3: (a) and (b) The buried state ($S_{Bu1}$) superimposed with previously reported crystal structures in PDB data bank (with 1U9I, 1TF7 colored in dark blue and medium blue, respectively).** The A-loop is shown as cartoons without transparency.

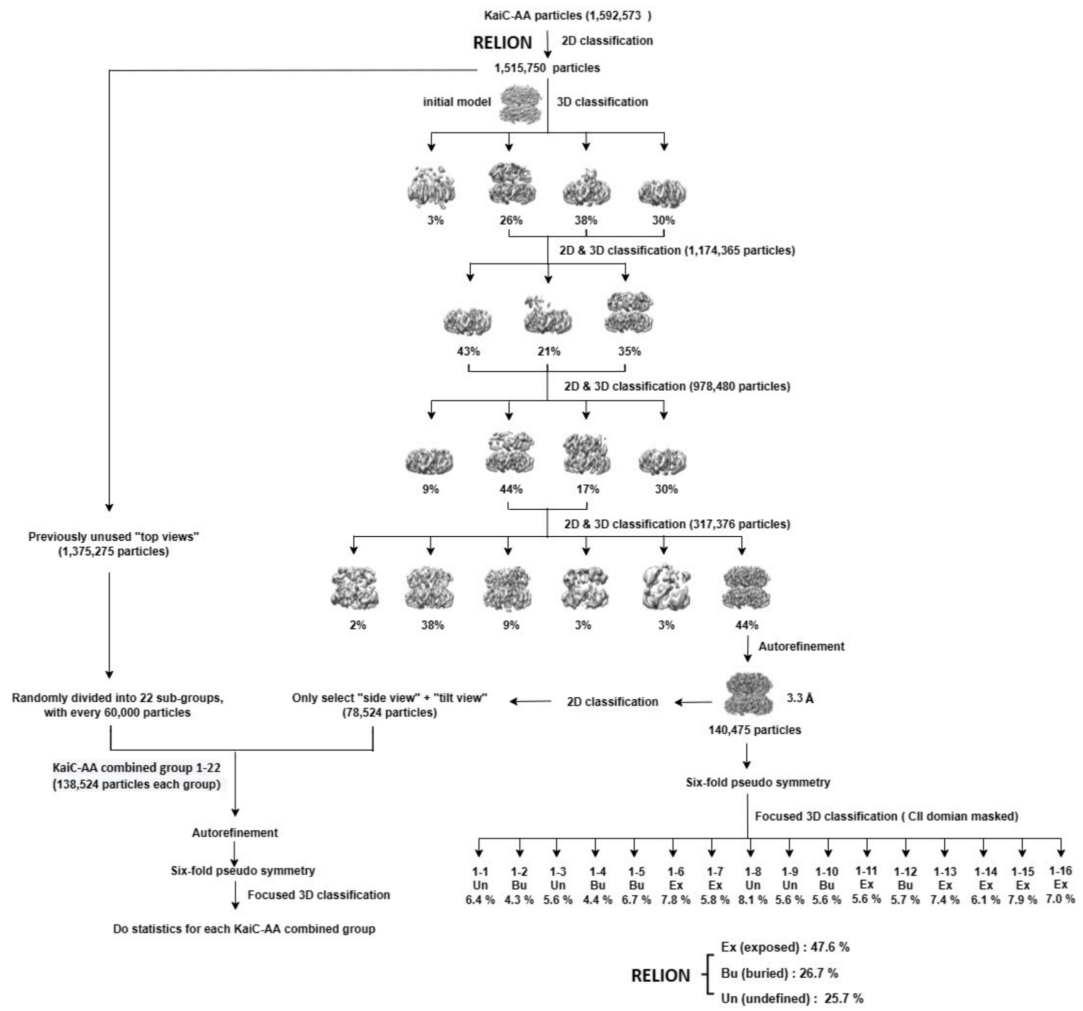


**Supplementary Figure 4: Data processing flow chart of KaiC-AA data set.** For the 3D classification results of the high quality well-defined structure (consist of 140,475 hexameric particles), there is 47.6% of subunits belong to the exposed state and 26.7% of subunits belong to the buried state, the rest 25.7% subunits remain unclassified. With *x*–*y* shift and angular information, we can put these exposed/buried subunits back into hexamers, and then carry out statistical analysis of the hexamer conformational patterns shown in Fig. 3a in the main text.

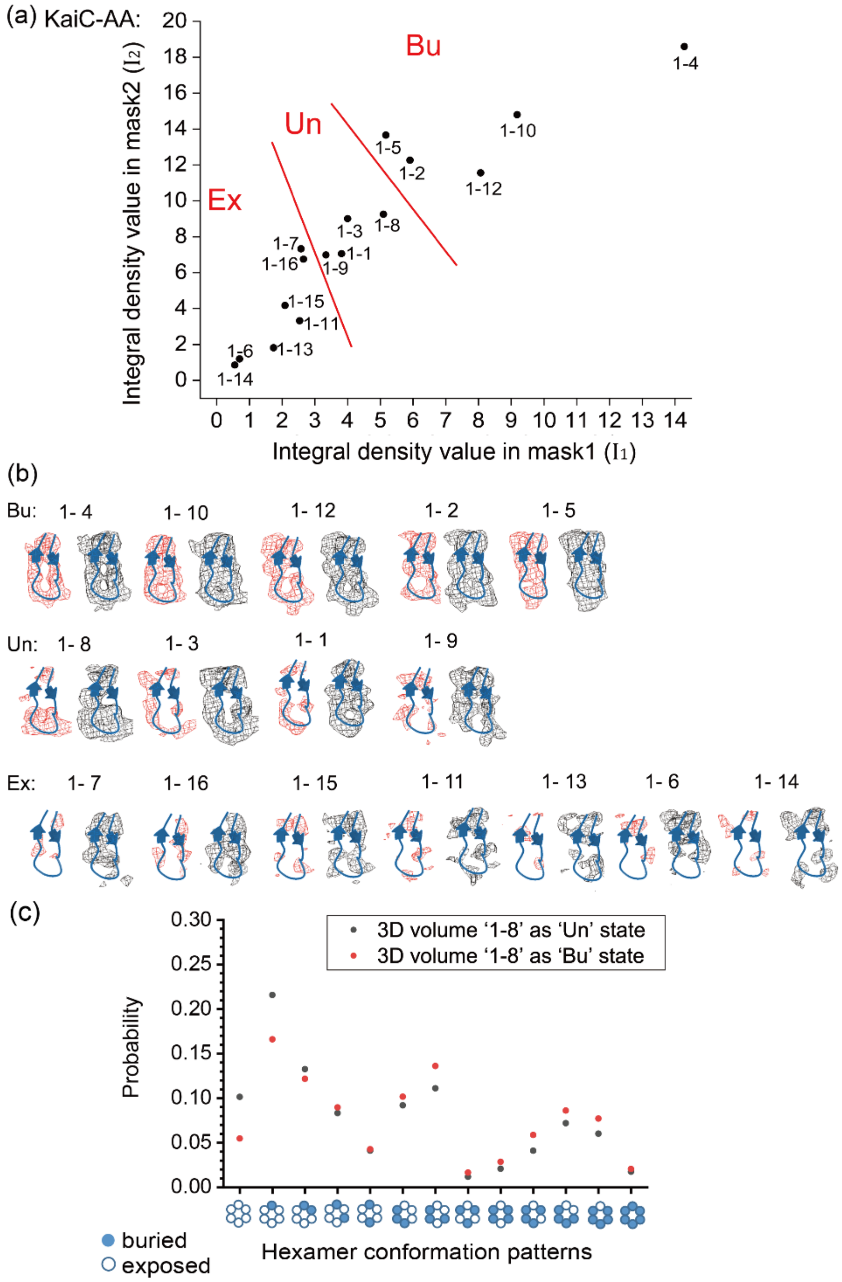


**Supplementary Figure 5: Criteria for distinguishing the exposed (Ex) state and buried (Bu) state in KaiC-AA. (a)** The overlap intensities (Integral density values) of 3D volumes classified by RELION (Supplementary Fig. 4) with the two masks that are used to characterize the buried state (Bu). **(b)** All 3D volumes (within the region corresponding to mask2) are shown at a high density threshold (4σ, red mesh, left) and a low density threshold (2σ, black mesh, right). The first row shows the structures that are classified as the buried (Bu) state. All structures in the first row agree with atomic model in all parts of the A-loop area at both the high and the low thresholds. The second row shows the undefined (Un) states, with density profiles that agree with the atomic model of the Bu state at the low threshold but not at the high threshold. The third row shows exposed (Ex) states, with the density profiles that disagree with the atomic model of the Bu state in the A-loop area at both the high and the low thresholds. **(c)** Comparison of hexamer conformation pattern distributions when 3D volume "1-8" is classified as "Un" (black dots) or "Bu" state (red dots).

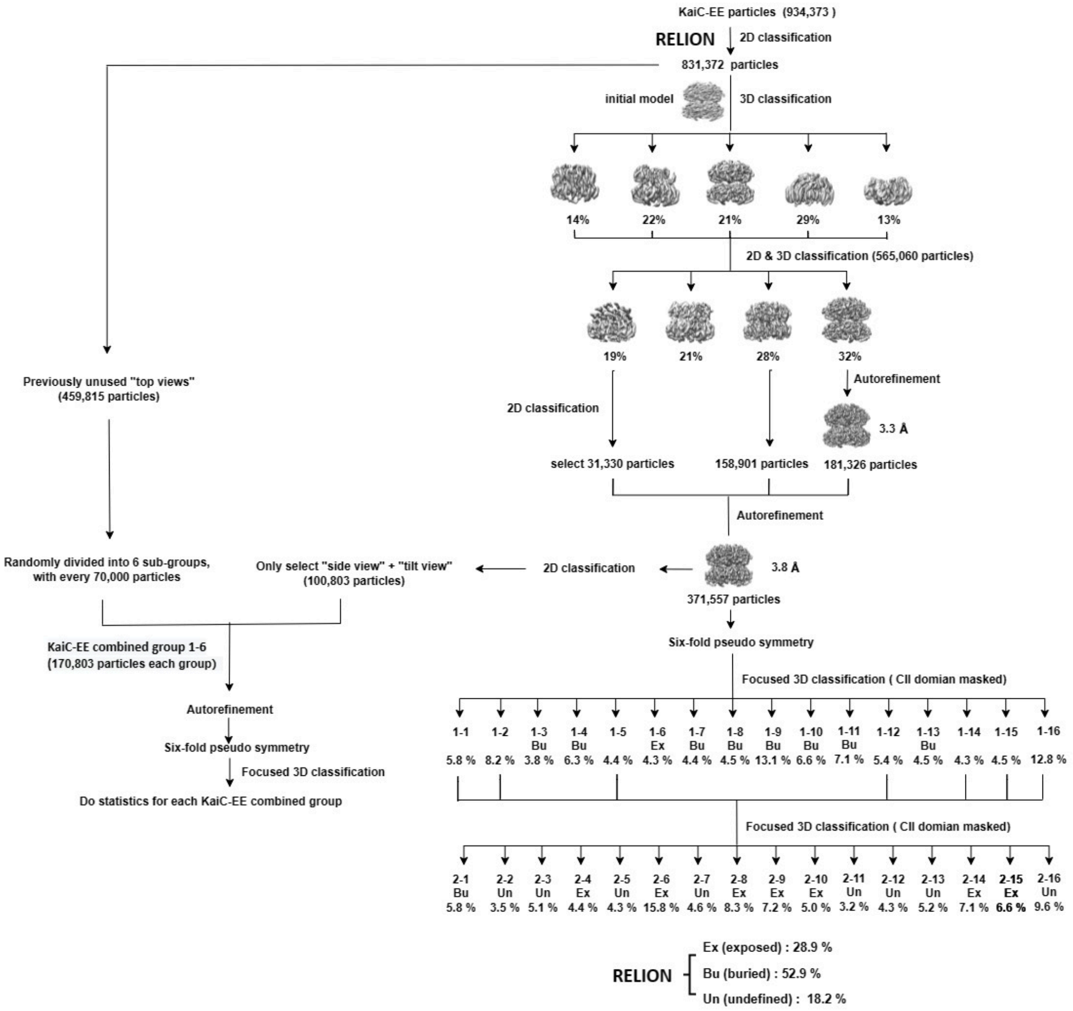


**Supplementary Figure 6: Data processing flow chart of KaiC-EE data set.** For the 3D classification results of the high quality well-defined structure (consist of 371,557 hexameric particles), there is 28.9% of subunits belong to the exposed state and 52.9% of subunits belong to the buried state, the rest 18.2% subunits remain unclassified. With *x*–*y* shift and angular information, we can put these exposed/buried subunits back into hexamers, and then carry out statistical analysis of the hexamer conformational patterns shown in Fig. 3b in the main text.

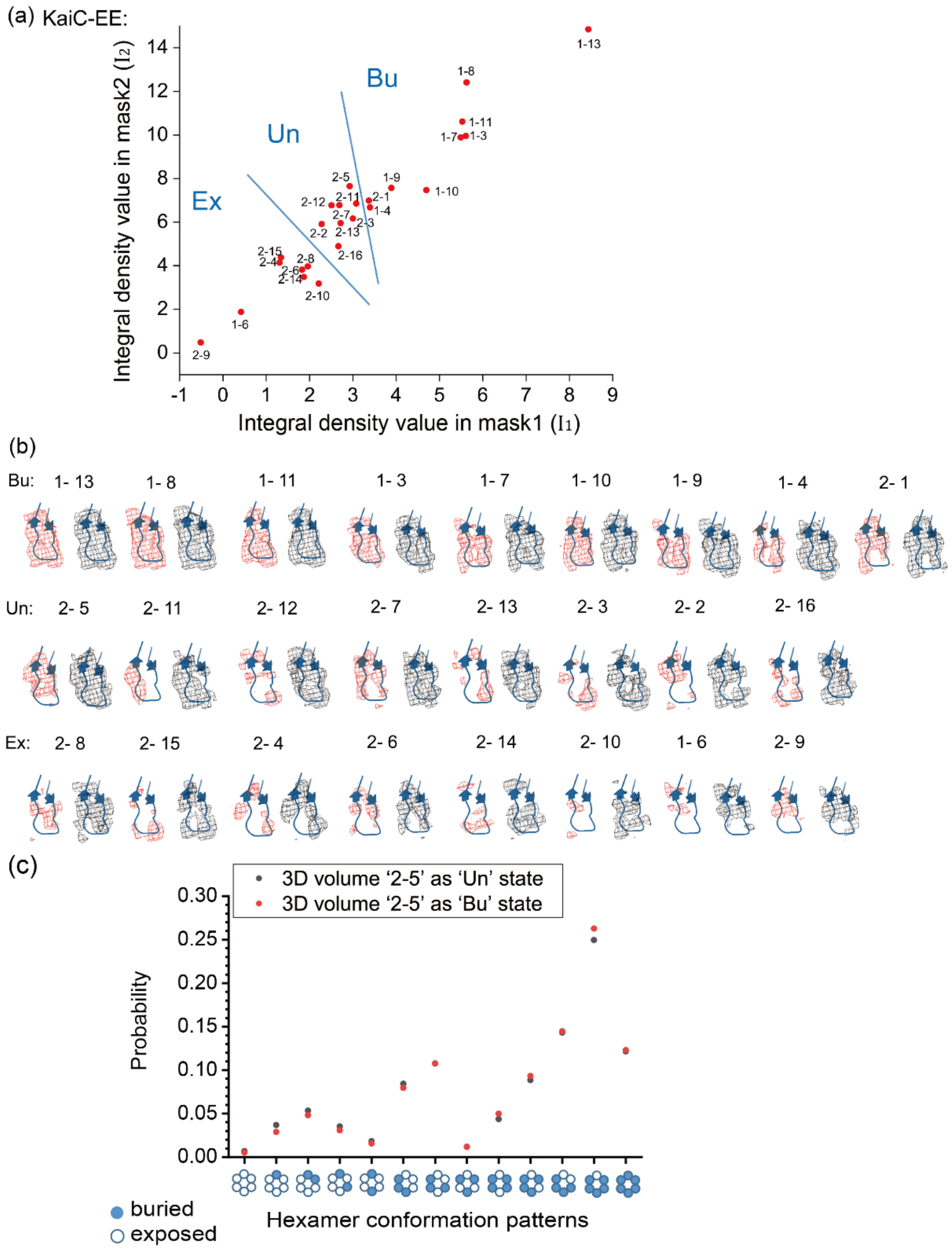


**Supplementary Figure 7: Criteria for distinguishing the exposed (Ex) state and buried (Bu) state in KaiC-EE. (a)** The overlap intensities (Integral density values) of 3D volumes classified by RELION (Supplementary Fig. 6) with the two masks that are used to characterize the buried state (Bu). **(b)** All 3D volumes (within the region corresponding to mask2) are shown at a high density threshold (4σ, red mesh, left) and a low density threshold (2σ, black mesh, right). The first row shows the buried (Bu) states, the second row shows undefined (Un) states, the third row shows exposed (Ex) states. Note that the 3D volume 2-5 has a poor resolution and weak density at the bottom at the 4σ density threshold, and the 3D volume 2-11 only has density on the left-side at the 4σ density threshold. So both 2-5 and 2-11, which are at the boundary of the Un states and the Bu states, are classified as undefined (Un). However, even including 2-5 as a buried state

does not affect the final results. **(c)** Comparison of hexamer conformation pattern distributions when 3D volume "2-5" is classified as "Un" (black dots) or "Bu" state (red dots).

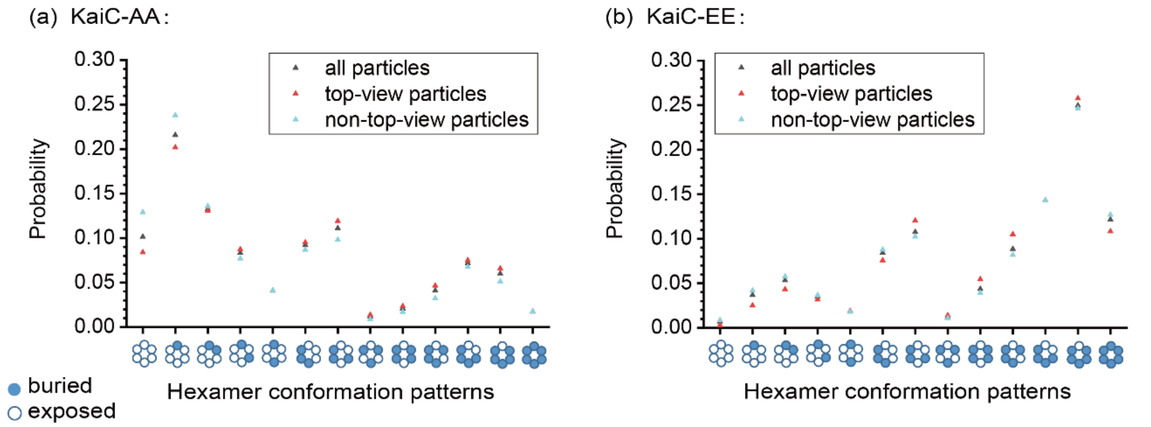


**Supplementary Figure 8:** Comparisons of the statistics of thirteen hexamer conformational patterns for the non-top-view particles only (light blue triangles), the top-view particles only (red triangles), and all particles (black triangles) for (a) Kai-AA particles; (b) Kai-EE particles.

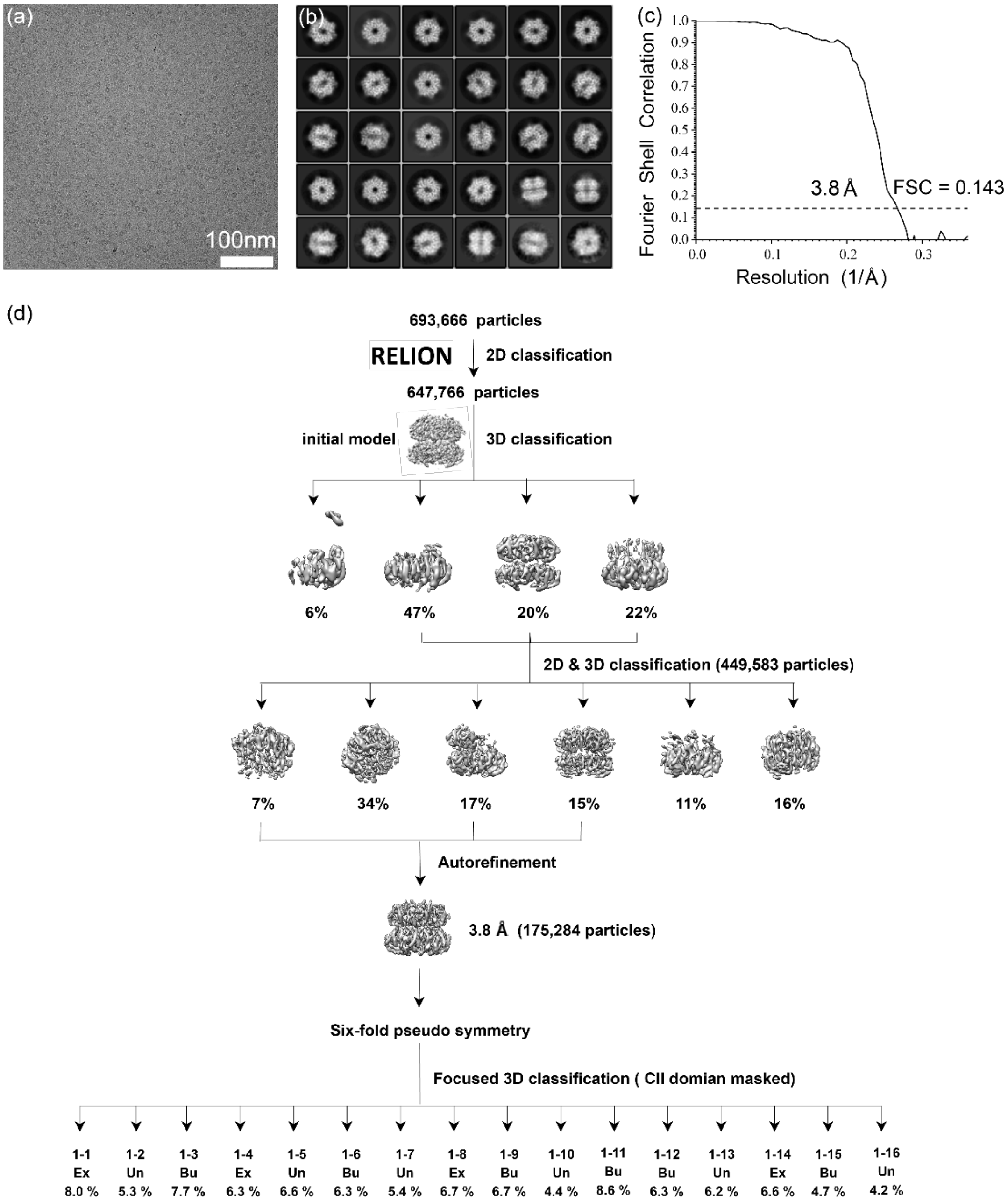


**Supplementary Figure 9: Cryo-EM structure determination and data processing flow chart of the mixed sample data set. (a)** Typical cryo-EM micrograph of the mixed sample data set, taken with a FEI Titan Krios G2 microscope equipped with the post-column Gatan BioQuantum energy filter connected to Gatan K2 Summit direct electron detector. Scale bar, 100 nm. **(b)** Gallery of unsupervised 2D class averages of the mixed sample data set. **(c)** Fourier shell correlation (FSC) for two independently refined halves of the mixed sample data set. During the refinement we imposed C6 symmetry. **(d)** Data processing flow chart of the mixed sample data set.

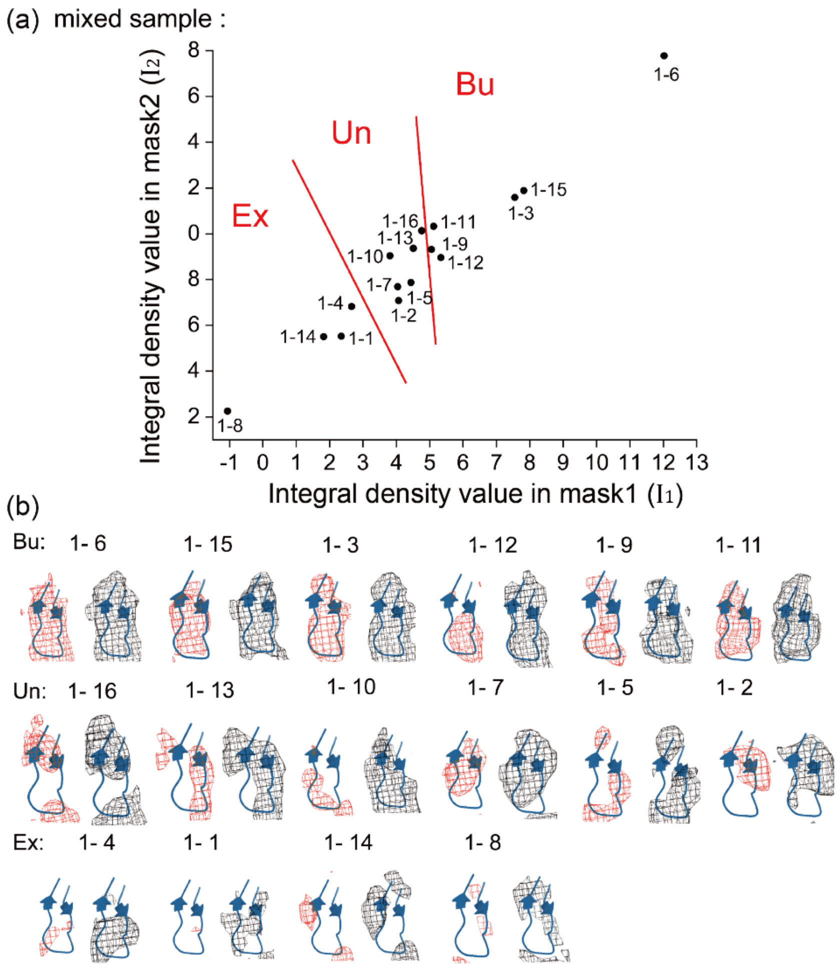


**Supplementary Figure 10: Criteria for distinguishing the exposed (Ex) state and buried (Bu) state in mixed sample. (a)** The overlap intensities (Integral density values) of 3D volumes classified by RELION (Supplementary Fig. 9) with the two masks that are used to characterize the buried state (Bu). **(b)** All 3D volumes (within the region corresponding to mask2) are shown at a high density threshold (4σ, red mesh, left) and a low density threshold (2σ, black mesh, right). The first row shows the buried (Bu) states, the second row shows the undefined (Un) states, the third row shows the exposed (Ex) states. Note that the 3D volume 1-16 and 1-13, which at the boundary between the Un state and the Bu state, have poor resolution and weak densities at the 4σ density threshold, so they are classified as undefined (Un).

## S2 Statistics of pattern distribution in pure hexamers

In this note, we briefly explain some critical points and introduce additional fitting methods and results mentioned in the main text, regarding the statistics of hexamer pattern distribution in pure hexamers.

**The conformational patterns of hexamer.** As mentioned in the main text, each monomer could be either in the exposed (Ex) state or buried (Bu) state, so there should be $2^6 = 64$ possible hexamer configurations in total. Because all the monomers are regarded identical, the configurations that only differ in a rotation operation can be grouped into the same hexamer conformational pattern, as is shown in Supplementary Fig. 11, and the number of configurations in the group is the pattern's degeneracy. Furthermore, for the two patterns that only differ in chirality (pattern-(7) and pattern-(8) in Supplementary Fig. 11a), we found that their numbers (in KaiC-AA, pattern-(7) has 1,292 hexameric particles pattern-(8) has 1,399 hexameric particles; in KaiC-EE, pattern-(7) has 6,306 hexameric particles pattern-(8) has 6,248 hexameric particles) are quite close. Hence, we ignore this chiral effect and regard the two patterns the same. So, there are 13 patterns in total, whose degeneracies are listed in Supplementary Fig. 11b.

**Statistics of the previously unused "top view" hexameric particles.** To avoid bias in particle selection, we also carried out statistical analysis by including a large number of top-view particles that were not used in the analysis reported in the main text, which include 1,375,275 KaiC-AA top-view particles and 459,815 KaiC-EE top-view particles, see Supplementary Figs. 4 and 6 for illustration of this additional analysis. These ``previously unused" top-view particles were randomly divided into sub-groups with equal number of particles (22 groups for KaiC-AA and 6 groups for KaiC-EE). Then, each sub-group of top-view particles were combined with all the non-top-view ("side view" and "tilt view") particles for KaiC-AA and KaiC-EE, respectively. These combined groups of particles are numbered from "KaiC-AA combined group 1" to "KaiC-AA combined group 22" (or from "KaiC-EE combined group 1" to "KaiC-EE combined group 6"). Finally, we performed statistical analysis for each combined group following the same procedures described in the main text by using RELION (Supplementary Figs. 4 and 6). We show the statistical results of two such combined groups for KaiC-AA and KaiC-EE respectively in Supplementary Fig. 12, which indicates that including these previously unused hexameric particles did not change statistics of the hexamer patterns significantly.

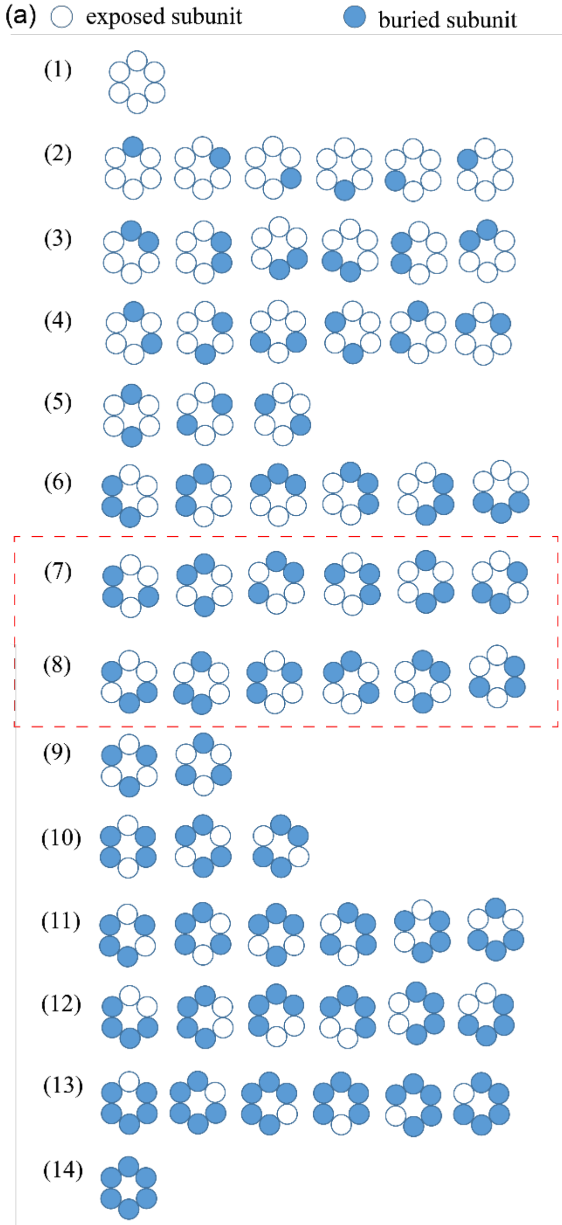


(b)

| pattern | the degeneracy $\Omega_k$ | $n_k$ |
|---|---|---|
| 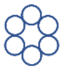 | $\Omega_1 = 1$ | $n_1 = 6$ |
| 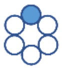 | $\Omega_2 = 6$ | $n_2 = 5$ |
| 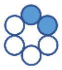 | $\Omega_3 = 6$ | $n_3 = 4$ |
| 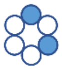 | $\Omega_4 = 6$ | $n_4 = 4$ |
| 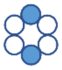 | $\Omega_5 = 3$ | $n_5 = 4$ |
| 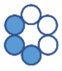 | $\Omega_6 = 6$ | $n_6 = 3$ |
| 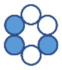 | $\Omega_7 = 12$ | $n_7 = 3$ |
| 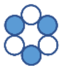 | $\Omega_8 = 2$ | $n_8 = 3$ |
| 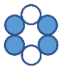 | $\Omega_9 = 3$ | $n_9 = 2$ |
| 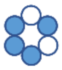 | $\Omega_{10} = 6$ | $n_{10} = 2$ |
| 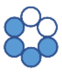 | $\Omega_{11} = 6$ | $n_{11} = 2$ |
| 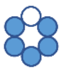 | $\Omega_{12} = 6$ | $n_{12} = 1$ |
| 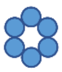 | $\Omega_{13} = 1$ | $n_{13} = 0$ |

**Supplementary Figure 11: Considering the structure degeneracy, 64 configurations can be combined into 13 conformational patterns. (a)** These 64 configurations are grouped into 13 conformational patterns according to the same pattern. Note that pattern (7) and (8) are only different in chirality, and our experiment shows very little difference between their numbers. So these two patterns are combined into one. **(b)** The degeneracy $\Omega_k$ and the number of exposed state $n_k$ are listed for each hexamer pattern.

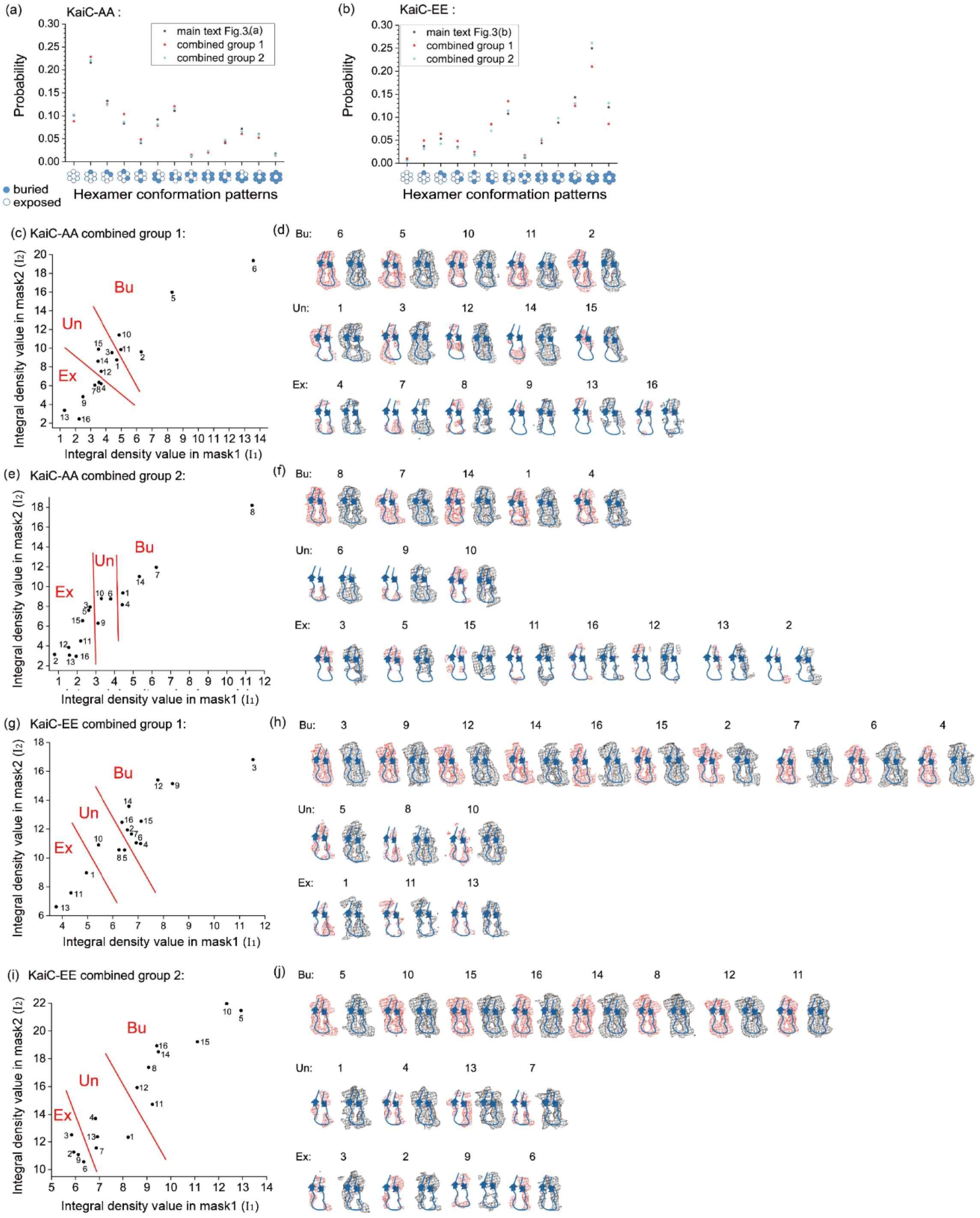


**Supplementary Figure 12: Including previously unused hexameric particles due to orientation preference does not change the statistical results. (a)** Comparison of the hexamer conformational pattern distribution in main text Fig. 3a (24,240 rings) and that of KaiC-AA combined group 1 (20,133 rings) and combined group 2 (21,638 rings). **(b)** Comparison of the hexamer conformational pattern distribution in main text Fig. 3b (116,785 rings) and that of KaiC-EE combined group 1 (84,226 rings) and combined group 2 (13,536 rings)). Probability of each data set was normalized. **(c-j)** The overlap intensities (Integral density values) of 3D

volumes classified by RELION with the two masks that are used to characterize the buried state (Bu) for KaiC-AA combined group 1 **(c)**, KaiC-AA combined group 2 **(e)**, KaiC-EE combined group 1 **(g)**, KaiC-EE combined group 2 **(i)**. All 3D volumes (within the region corresponding to mask2) are shown at a high density threshold (4σ, red mesh, left) and a low density threshold (2σ, black mesh, right) for KaiC-AA combined-group 1 **(d)**, KaiC-AA combined-group 2 **(f)**, KaiC-EE combined-group 1 **(h)**, KaiC-EE combined-group 2 **(j)**.

## S3 Statistics of pattern distribution in mixed hexamers

In this note, we describe some details about conformational statistics for mixed hexamers.

**Conformational pattern distribution and monomer arrangement for mixed hexamers.** In principle, the conformational pattern distribution for mixed hexamers should lie between the two pure cases. This is because the mixed hexamers can have 14 distinct monomer arrangements (see Supplementary Table 2), though the arrangement distribution $q_l$ is unknown. Two extreme scenarios to infer $q_l$ is assuming either monomers are: 1) fully mixed, i.e., AA and EE monomers appear in the ring with no preference (fully-mixed scenario), or 2) not mixed at all (the no-mixing scenario). In fully-mixed scenario, $q_l$ can be calculated analytically and listed in Supplementary Table 2. In no-mixing scenario, only the two pure arrangements have non-zero $q_l$. With the two assumptions of $q_l$, we can calculate the theoretical distribution of hexamer patterns in respective scenarios. These two theoretical distributions are shown in Supplementary Fig. 13, with the three experimental distribution (two pure cases and the mixing case) also presented for comparison. It appears that neither of the two extreme scenarios can well explain the experimental distribution of mixed hexamers.

**The fitting for subunit arrangement.** Generally, $q_l$ could be obtained from fitting. We fit the theoretical predicted $P_k$ (according to Eqs. 5 & 6 in main text) to experimental observation ($P_{k(ex)}$) by using the following least square method:

$$min_q\left(\sum_{k=1}^{13}\left(\sum_{l=1}^{14} q_l p_l(k) - P_{k\ (ex)}\right)^2 + \lambda\left(\sum_{l=1}^{14} q_l(1-E_l) - \sum_{l=1}^{14} q_l E_l\right)^2\right), \quad (1)$$

subject to the constraints: $\sum_{l=1}^{14} q_l = 1,\ 0 \le q_l \le 1$. The second term above is introduced to enforce the overall equal percentage of KaiC-EE and KaiC-AA monomers with $E_l$ the percentage of KaiC-EE monomer in the hexamer with arrangement-$l$, and λ is a weight constant. The fitting results $q_l$ and performance $R^2$ are in general insensitive to λ (Supplementary Figs. 14 and 15b). We choose a specific $\lambda = 0.1$ in the analysis in main text to enforce the difference between KaiC-AA and KaiC-EE percentage to be less than 10%.

From the fitting result of $q_l$ shown in Supplementary Fig. 15a, in the absence of coupling, the resulting distribution is almost the same as no-mixing scenario, which is inconsistent with our previous results and certainly not the case. Hence strong coupling is necessary.

**The extended Ising model for mixed hexamers.** We extended the original Ising model by considering the dependence of the coupling constant $J$ on whether the two neighboring monomers are the same or not. Specifically, we modified our original Ising model by introducing the composition-dependent coupling constant $\Delta J$ into Eq. (4) introduced in the main text. Specifically, the modified Hamiltonian has the following form:

$$H_l\left(\vec{S}\right) = -\sum_{<ij>} J_{ij} S_i S_j - \sum_i [B_{AA}(1-\sigma_{i,l}) + B_{EE}\sigma_{i,l}] S_i \, ,$$

with

$$J_{ij} = J + \Delta J \times \frac{(2\sigma_i - 1)(2\sigma_j - 1) - 1}{2},$$

where $\Delta J$ is the difference in coupling constant between the same type of monomers (EE-EE or AA-AA) and different types of monomers (AA-EE) and J is the coupling constant for pure hexamers.

The monomer arrangements are assumed to be random so each monomer in the ring has an equal probability of being AA or EE, i.e., corresponding to the fully-mixed scenario mentioned in the main text. Supplementary Figure 17 shows how $R^2$ of the fitting depends on $\Delta J$. The best fit corresponds to $\Delta J = 0.09 > 0$, which is consistent with the observation that the overall (averaged) coupling constant in the mixed hexamer is smaller than that for the pure hexamers: $J_{mix} < J$. However, the optimal $R^2 \approx 0.83$, which indicates that the improvement is quite limited. Therefore, introducing a different coupling strength between EE and AA is not enough to explain the data for mixed hexamers.

| subunit arrangements | $q_l$ | fully-mixed | no-mixing |
|---|---|---|---|
| AAAAAA | $q_1$ | 0.0156 | 0.5 |
| AAAAAE | $q_2$ | 0.0938 | 0 |
| AAAAEE | $q_3$ | 0.0938 | 0 |
| AAAEAE | $q_4$ | 0.0938 | 0 |
| AAEAAE | $q_5$ | 0.0469 | 0 |
| AAAEEE | $q_6$ | 0.0938 | 0 |
| AAEAEE | $q_7$ | 0.0938 | 0 |
| AAEEAE | $q_8$ | 0.0938 | 0 |
| AEAEAE | $q_9$ | 0.0313 | 0 |
| AAEEEE | $q_{10}$ | 0.0938 | 0 |
| AEAEEE | $q_{11}$ | 0.0938 | 0 |
| AEEAEE | $q_{12}$ | 0.0469 | 0 |
| AEEEEE | $q_{13}$ | 0.0938 | 0 |
| EEEEEE | $q_{14}$ | 0.0156 | 0.5 |

**Supplementary Table 2**: Enumeration of subunit arrangements. The arrangements for the two extreme scenarios are also listed.

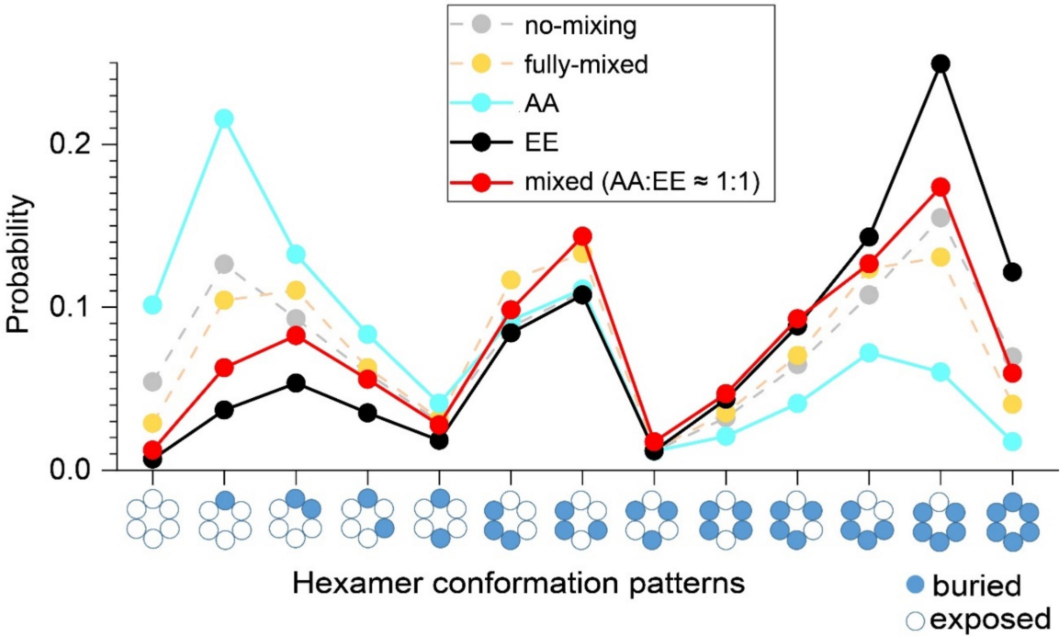


**Supplementary Figure 13:** Hexamer conformation pattern distributions, predicted in two extreme scenarios (connected by dotted line) and measured in three experimental conditions (connected by solid line). Predicted distribution in no-mixing scenario and fully-mixed scenario are connected by gray dotted line and yellow dotted line, respectively. Experimental distribution of pure KaiC-AA, pure KaiC-EE and the mixed sample are connected by cyan solid line, black solid line and red solid line, respectively. The conformation distribution of mixed hexamers lies between two pure distributions, but can be explained neither by no-mixing assumption nor by fully-mixing assumption.

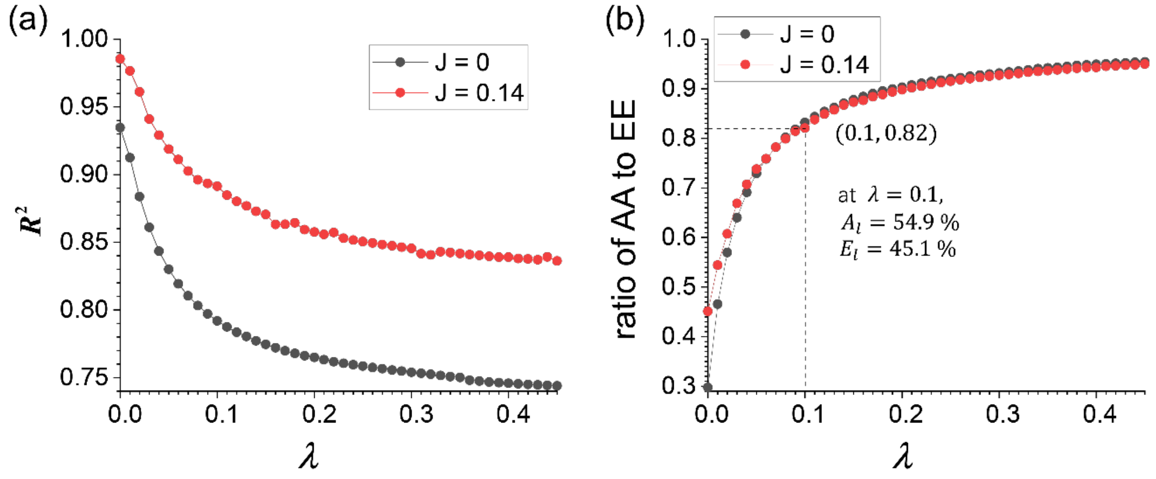


**Supplementary Figure 14: (a)** $R^2$ versus $\lambda$ when the coupling constant $J = 0$ (black) or $J = 0.14$ (red). **(b)** The ratio of AA monomer to EE monomer for the fitted arrangement distribution varies with $\lambda$ when the coupling constant $J = 0$ (black) or $J = 0.14$ (red). The fitting performance is quite robust when $\lambda$ varies.

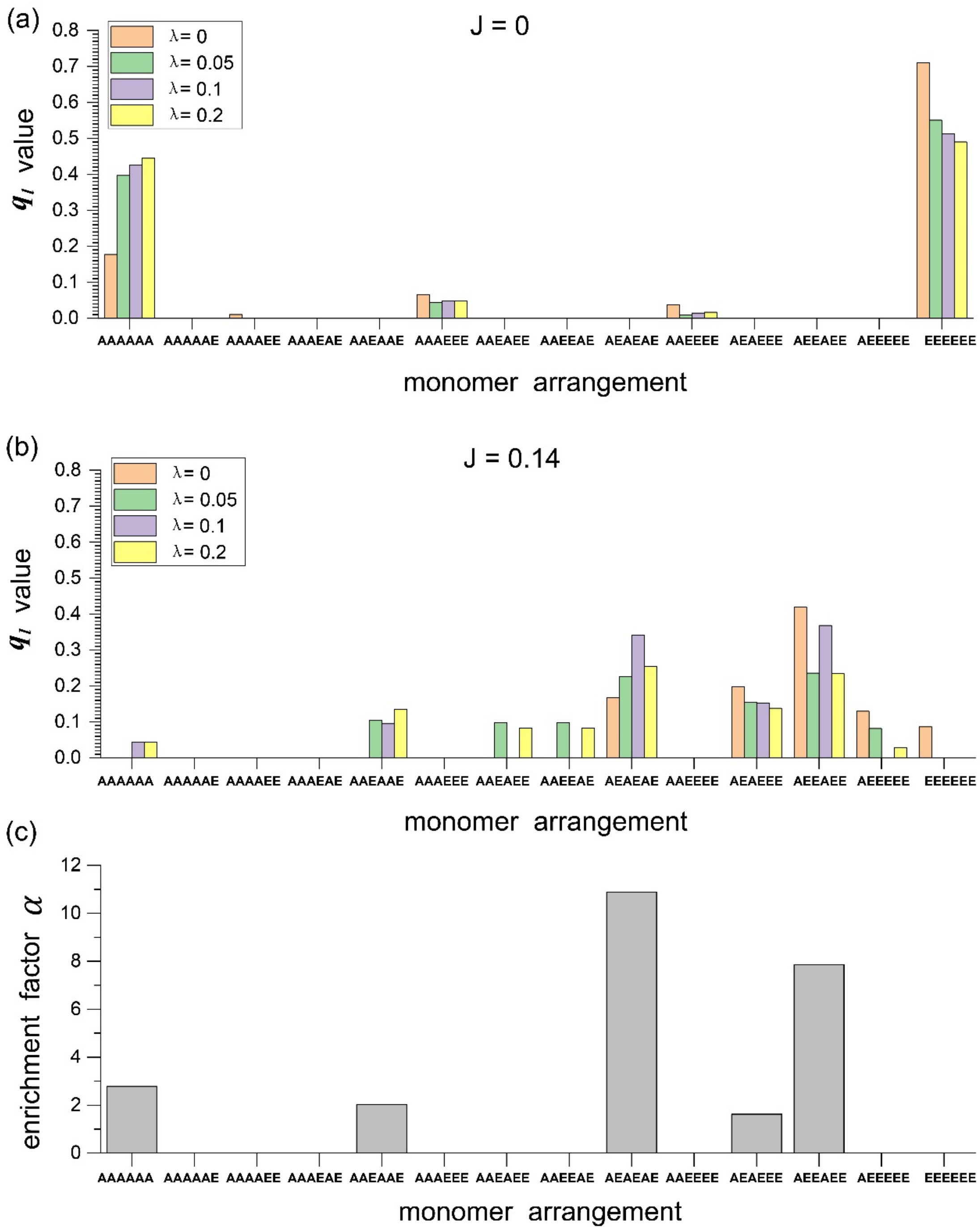


**Supplementary Figure 15: The monomer arrangement distribution with different choice of λ in the case that coupling constant (a) $\mathrm{J} = \mathbf{0}$ or (b) $\mathrm{J} = \mathbf{0.14}$.** The distribution of $q_l$ is insensitive to λ. In the absence of coupling ($\mathrm{J} = 0$), the fitting result tells that only pure hexamers exist, which is inconsistent with previous results and definitely not the real case, so the presence of coupling is necessary. **(c)** The enrichment factor α (the ratio of optimized $q_l$ values when $\lambda = 0.1$ in (b) to that under fully-mixed scenario (Table S2)) of 14 monomer arrangements. It shows that α >> 1 with the monomer arrangement 'AEAEAE', which indicate that there is a higher probability for two neighboring subunits to have different phosphorylation levels.

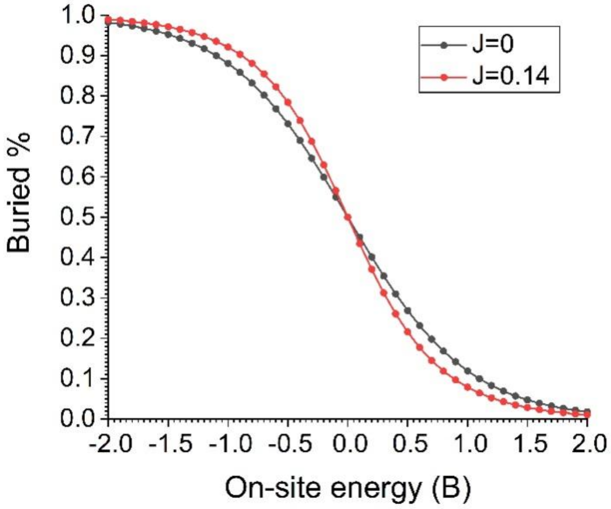


**Supplementary Figure 16**: The percentage of buried state as we vary the on-site energy ($B$) for without coupling ($J = 0$) and with coupling ($J = 0.14$).

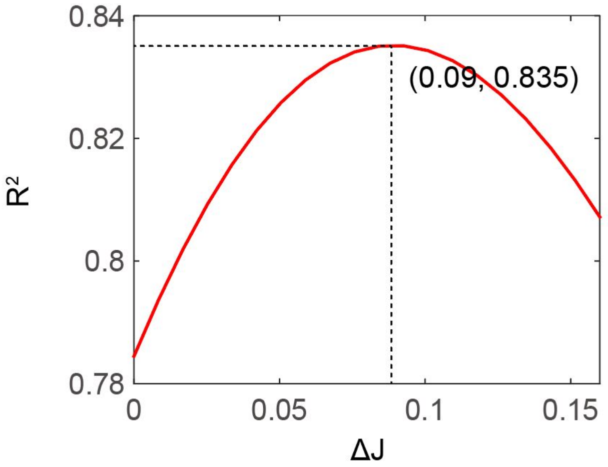


**Supplementary Figure 17**: The dependence of $R^2$ on $\Delta J$ in extended Ising model in the fully-mixed scenario.
