# Supplementary material for: Determining subunit-subunit interaction from statistics of cryo-EM images: observation of nearest-neighbor coupling in a circadian clock protein complex
Source: Nat Commun. 2023 Sep 22;14:5907. doi: 10.1038/s41467-023-41575-1 (PMC10516925; doi:10.1038/s41467-023-41575-1)
Supplement: Supplementary file 3 — Reporting Summary [file 41467_2023_41575_MOESM3_ESM.pdf]

nature portfolio

Corresponding author(s): Michael Rust, Yuhai Tu, Qi Ouyang

Last updated by author(s): 2023/09/04

Reporting Summary

Nature Portfolio wishes to improve the reproducibility of the work that we publish. This form provides structure for consistency and transparency in reporting. For further information on Nature Portfolio policies, see our Editorial Policies and the Editorial Policy Checklist.

Statistics

For all statistical analyses, confirm that the following items are present in the figure legend, table legend, main text, or Methods section.

n/a	Confirmed	
☐	☒	The exact sample size (*n*) for each experimental group/condition, given as a discrete number and unit of measurement
☐	☒	A statement on whether measurements were taken from distinct samples or whether the same sample was measured repeatedly
☒	☐	The statistical test(s) used AND whether they are one- or two-sided<br>*Only common tests should be described solely by name; describe more complex techniques in the Methods section.*
☒	☐	A description of all covariates tested
☐	☒	A description of any assumptions or corrections, such as tests of normality and adjustment for multiple comparisons
☐	☒	A full description of the statistical parameters including central tendency (e.g. means) or other basic estimates (e.g. regression coefficient) AND variation (e.g. standard deviation) or associated estimates of uncertainty (e.g. confidence intervals)
☒	☐	For null hypothesis testing, the test statistic (e.g. *F*, *t*, *r*) with confidence intervals, effect sizes, degrees of freedom and *P* value noted<br>*Give P values as exact values whenever suitable.*
☒	☐	For Bayesian analysis, information on the choice of priors and Markov chain Monte Carlo settings
☒	☐	For hierarchical and complex designs, identification of the appropriate level for tests and full reporting of outcomes
☒	☐	Estimates of effect sizes (e.g. Cohen's *d*, Pearson's *r*), indicating how they were calculated

Our web collection on statistics for biologists contains articles on many of the points above.

Software and code

Policy information about availability of computer code

Data collection	MotionCor2 (https://emcore.ucsf.edu/ucsf-software); and Gctf (https://github.com/Characterisation-Virtual-Laboratory/Cryo-EM-Processing-Tool/blob/master/userGuide.md)
Data analysis	RELION, see https://relion.readthedocs.io/en/release-4.0/

For manuscripts utilizing custom algorithms or software that are central to the research but not yet described in published literature, software must be made available to editors and reviewers. We strongly encourage code deposition in a community repository (e.g. GitHub). See the Nature Portfolio guidelines for submitting code & software for further information.

Data

Policy information about availability of data

All manuscripts must include a data availability statement. This statement should provide the following information, where applicable:

- Accession codes, unique identifiers, or web links for publicly available datasets
- A description of any restrictions on data availability
- For clinical datasets or third party data, please ensure that the statement adheres to our policy

The three-dimensional cryo-EM density maps of KaiC-AA and KaiC-EE are deposited into the Electron Microscopy Data Bank (EMDB) KaiC-AA: EMD-32952, KaiC-EE: EMD-32953. The corresponding atomic coordinates are deposited in the Protein Data Bank (PDB) KaiC-AA: 7X1Y, KaiC-EE: 7X1Z.

Research involving human participants, their data, or biological material

Policy information about studies with human participants or human data. See also policy information about sex, gender (identity/presentation), and sexual orientation and race, ethnicity and racism.

Reporting on sex and gender	NA, our study is on a bacterial protein complex.
Reporting on race, ethnicity, or other socially relevant groupings	NA, our study is on a bacterial protein complex.
Population characteristics	NA, our study is on a bacterial protein complex.
Recruitment	NA, our study is on a bacterial protein complex.
Ethics oversight	NA, our study is on a bacterial protein complex.

Note that full information on the approval of the study protocol must also be provided in the manuscript.

Field-specific reporting

Please select the one below that is the best fit for your research. If you are not sure, read the appropriate sections before making your selection.

☒ Life sciences ☐ Behavioural & social sciences ☐ Ecological, evolutionary & environmental sciences

For a reference copy of the document with all sections, see nature.com/documents/nr-reporting-summary-flat.pdf

Life sciences study design

All studies must disclose on these points even when the disclosure is negative.

Sample size	We aim to have much high quality data as possible for good statistics. We were able to achieve this goal with 1592573 KaiC-AA hexameric particles; 934,373 KaiC-EE particles; and 693,666 hexameric particles of the mixed hexameric particles, which are sufficient for us to carry out accurate statistical studies of the distributions of different conformational states.
Data exclusions	No data was excluded.
Replication	The KaiC hexamer structure was measured for many KaiC hexamer particales (1592573 KaiC-AA hexameric particles; 934,373 KaiC-EE particles; 693,666 hexameric particles of the mixed hexameric particles), and these replicated structures are consistent with each other.
Randomization	The Cryo-EM images are grouped by their orientations (top-view, side-view, tilt-view) and then randomly grouped together to obtain the averaged 3D-structure, which does not depend on the specific random grouping of the Cryo-EM images.
Blinding	The grouping is random and the results are independent of the specific grouping.

Reporting for specific materials, systems and methods

We require information from authors about some types of materials, experimental systems and methods used in many studies. Here, indicate whether each material, system or method listed is relevant to your study. If you are not sure if a list item applies to your research, read the appropriate section before selecting a response.

Materials & experimental systems

n/a	Involved in the study	
☒	☐	Antibodies
☒	☐	Eukaryotic cell lines
☒	☐	Palaeontology and archaeology
☒	☐	Animals and other organisms
☒	☐	Clinical data
☒	☐	Dual use research of concern
☒	☐	Plants

Methods

n/a	Involved in the study	
☒	☐	ChIP-seq
☒	☐	Flow cytometry
☒	☐	MRI-based neuroimaging
